# Toward Accurate Extraction of Respiratory Frequency From the Photoplethysmogram: Effect of Measurement Site

**DOI:** 10.3389/fphys.2019.00732

**Published:** 2019-06-18

**Authors:** Vera Hartmann, Haipeng Liu, Fei Chen, Wentao Hong, Stephen Hughes, Dingchang Zheng

**Affiliations:** ^1^ School of Allied Health, Faculty of Health, Education, Medicine, and Social Care, Anglia Ruskin University, Chelmsford, United Kingdom; ^2^ Department of Electrical and Electronic Engineering, Southern University of Science and Technology, Shenzhen, China

**Keywords:** photoplethysmography, photoplethysmographic measurement site, multi-site photoplethysmography, breathing pattern, respiratory frequency measurement

## Abstract

**Background:** It is known that the respiration-modulated photoplethysmographic (PPG) signals could be used to derive respiratory frequency (RF) and that PPG signals could be measured from different body sites. However, the accuracy of RF derived from PPG signals of different body sites has not been comprehensively investigated.

**Objective:** This study aims to investigate the difference in the accuracy of PPG-derived RFs between measurements from different body sites, respectively, for normal and deep breathing conditions.

**Methods:** Under normal and deep breathing patterns, the PPG signals were recorded sequentially in a randomized order from six body sites [finger, wrist under (anatomically volar), wrist upper (dorsal), earlobe, and forehead] of 36 healthy subjects. Simultaneously, the reference respiratory signal was measured by a respiratory belt on the chest. Using the frequency demodulation approach, respiratory signals were extracted from PPG signals for calculating RF by power spectral density. The bias between PPG-derived and reference RFs was then analyzed statistically using analysis of variance and non-parametric tests, Bland-Altman analysis, and linear regression to investigate the difference in RF bias between different sites.

**Results:** The RF bias was significantly influenced by the breathing pattern and measurement site (both *p* < 0.001). Under normal breathing, the RF bias was insignificant in the arm, forehead, and wrist under (all *p* > 0.05) and significant in the other sites (all *p* < 0.05). Significant linear relationship between PPG-derived and reference RFs existed at all the sites (*p* < 0.001) except the wrist upper (*p* > 0.05). The linearity between PPG-derived and reference RFs was highest at the forehead (slope of best-fit line: 0.90, *R*^2^: 0.64), followed by the earlobe, finger, arm, and wrist under (slope: 0.71, *R*^2^: 0.40). Under deep breathing, there was no significant RF bias in all the measurement sites (*p* > 0.05) except forehead (*p* = 0.048). The effect of measurement site on RF bias was not significant (*p* > 0.05). The finger had the smallest RF bias and the narrowest limits of agreement.

**Conclusion:** This study has demonstrated that the accuracy of PPG-derived RF depends on the measurement site and breathing pattern. The best sites are the forehead and finger, respectively, for normal and deep breathing patterns.

## Introduction

Measurement of vital sign of respiratory rate is clinically important. Respiratory frequency (RF) is defined as the reciprocal of respiratory period length. RF changes with the metabolic and cardiovascular conditions. Physiologically, RF reflects the cardiorespiratory changes during various physical activities (Nicolò et al., 2017). Pathologically, RF could be used to differentiate the severities of patients (Massaroni et al., 2018) and indicate the condition of recovery from anesthesia (Hochhausen et al., 2018) and the necessity of admission to the intensive care unit (Al-Khalidi et al., 2011). Despite its clinical significance, the clinical RF monitoring still relies on the manual recording, which is inaccurate and often neglected (Flenady et al., 2017).

The traditional automatic measurement of RF is contact-based, which requires big devices such as the spirometry, capnotmetry, and impedance pneumography (Shen et al., 2017). In recent years, many innovative devices have been attempted to continuously measure RF in non-contact ways. They are mainly based on the radar (Van Loon et al., 2016), body movement imaging (Massaroni et al., 2018), or thermal imaging (Cho et al., 2017). However, the majority of these methods are at the research and development stage, requiring further clinical validations.

Photoplethysmography (PPG) is a clinically validated and low-cost optical measurement technique, which is used to reflect the blood volumetric changes in the microvascular bed. PPG signals can be measured by the transmission and reflection methods by detecting the light transmitted through or reflected by the tissue. Transmission method is applicable on body sites with thin tissue such as fingertips or earlobe, while reflection method is applicable on most of body sites. The PPG waveforms differ in various body sites along propagation of the pulses from the heart distally to the peripheral ends such as the fingertips and toes (Lindholm et al., 2007). A typical PPG signal includes the quasi-constant and pulsatile components. The pulsatile component (0.6–2.0 Hz) mainly reflects the periodic blood volume changes within each cardiac cycle, and the quasi-constant component is associated with the respiratory (0.15–0.60 Hz), myogenic (0.052–0.15 Hz), neurogenic (0.021–0.052 Hz), and endothelial (0.0095–0.021 Hz) activities (Bentham et al., 2018). Therefore, respiratory rate or RF could be extracted from the recorded PPG signals using signal processing techniques.

In practice, RF is commonly estimated from the PPG signals by techniques in association with amplitude modulation (AM), frequency modulation (FM), and baseline wandering (BW). The FM method is based on the respiratory sinus arrhythmia, which causes heart rate to increase during inspiration and decrease during exhalation therein RF could be derived (Charlton et al., 2017). FM has also been widely used in deriving RF from PPG signal, with its validation results in good agreement with reference measurements (Touw et al., 2017).

Several studies have reported the differences in PPG-derived respiratory signal features between different body sites. The PPG-derived respiratory signal was found to be more than 10 times stronger in the head than the finger (Shelley et al., 2006). Additionally, respiratory signals of higher quality were extracted by the FM from the finger PPG in comparison with ear PPG (Charlton et al., 2017). The PPG signal from the head was found to be more sensitive to the fluctuations of blood oxygen saturation than those from ear and finger (Bebout and Bednarski, 2017). However, there has not been a comprehensive study to compare the effectiveness of extracting RF from PPG signal between different body sites.

The breathing patterns might also influence the effectiveness of RF extraction by fluctuating oxygen saturation. It has been reported that the correlation coefficients between PPG signals derived from finger and wrist were different when the measurements were performed under the normal and deep breathing patterns (Keikhosravi and Zahedi, 2013). The fluctuation of respiration-related PPG signal tends to be larger under deep breathing than normal breathing (Yuan et al., 2018). Therefore, it is important to consider the effect of breathing pattern in comparing the PPG-derived RFs between different body sites.

This study aimed to quantitatively investigate the effects of measurement site and breathing pattern on the measurement accuracy of the PPG-derived RF.

## Materials and Methods

### Subjects

Thirty-six healthy subjects (24 females and 12 males) without any known cardiovascular diseases participated in the study with written informed consent. The protocol was approved by the Research Ethics Committee of the Faculty of Medical Science, Anglia Ruskin University, UK. Table 1 gives an overview of basic subject information, including age, weight, height, and blood pressures.

**Table 1:** Basic clinical subject information including age, weight, height, and blood pressures.

Subject information				
No. subjects	36				
No. male	12				
No. female	24				
		**Mean**	**Min**	**Max**	**SD**
Age (years)		33	19	58	12
Weight (kg)		70	45	90	12
Height (cm)		170	154	186	8
SBP (mmHg)		117	93	172	14
DBP (mmHg)		77	58	98	9

### Measurement Procedure

**Figure 1:**
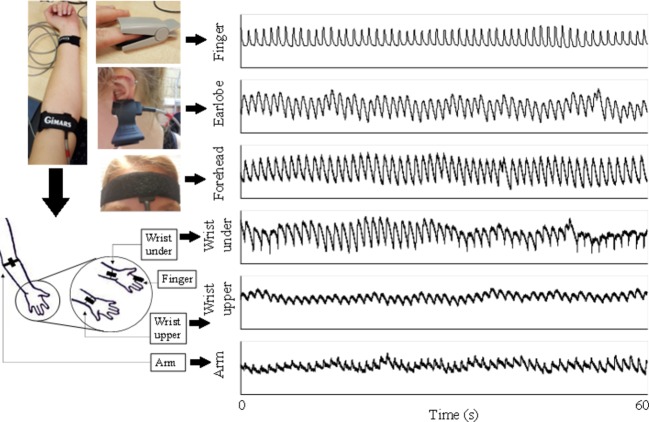
PPG signals recorded from six body sites of a subject under normal breathing. Written informed consent was obtained from the individual for the publication of this image.

The experiments were performed in a quiet measurement room at Anglia Ruskin University. After a 10-min sedentary relaxation, resting blood pressures (BPs) were measured using a clinically validated automatic BP monitor (HEM-7322U-E from Omron healthcare) (Table 1). Subsequently, the subjects were asked to lie down in supine position on a clinical bed, in order to keep a steady breathing pattern during measurement. A reflective PPG sensor was used in this study. The sensor was developed with an identical pair of surface-mount emitting diode (SME 2470-001, Honeywell) and photodiode (SMD 2420-001, Honeywell). The SME2470 is a high intensity aluminum gallium arsenide infrared emitting diode, which has a beam angle of 24°. The output peak wavelength of the emitting diode is about 880 nm, which matches with the maximum photosensitivity wavelength of the SMD2420 photodiode and supplies optimum optical characteristics and efficient optical coupling. The reflective PPG sensor was sequentially placed on different body sites (finger, wrist volar side, wrist dorsal side, arm, earlobe, and forehead) in a randomized order. The PPG measurement sites on the volar and dorsal sides of the wrist were referred to as simply as “wrist under” and “wrist upper” below. The same optical sensor was used for all the sites. To fix the PPG sensors, the finger clip, ear clip, and Velcro fastener were used on the finger, ear, and other sites (Figure 1). The reference respiration signal was measured by a strain gauge chest strap to detect the chest wall movement.

The PPG waveform and reference respiratory signal were digitally and simultaneously recorded with the sampling rate of 2,000 Hz by the data acquisition system MP160 using the software Biopac ACQKnowledge 5.0. Each recording from a certain measurement site started after the PPG waveform and reference respiratory signal were clearly displayed on the screen. Each recording lasted for 1 min. There was a 1-min gap between recordings. The measurement order between different sites was randomized. During the whole measurement, the participants were asked not to talk or move to reduce the potential effect of motion artifacts on the quality of PPG signals.

Considering the possible effect of breathing pattern on the estimation accuracy of PPG-derived RF, the PPG recordings from different measurement sites were performed under both normal and deep breathing patterns. Under normal breathing pattern, the subject was relaxed and breathed under a normal pace. Deep breathing was fulfilled by following a paced breathing app (Paced breathing, Trex LLC) with a defined inhalation and exhalation time of 5 s, respectively. In total, 12 recordings of PPG signals and 12 corresponding reference respiratory signals were obtained from each subject (from six body sites and two breathing patterns).

### Respiratory Frequency Extraction From Photoplethysmographic Signal and Reference Respiratory Signal

**Figure 2:**
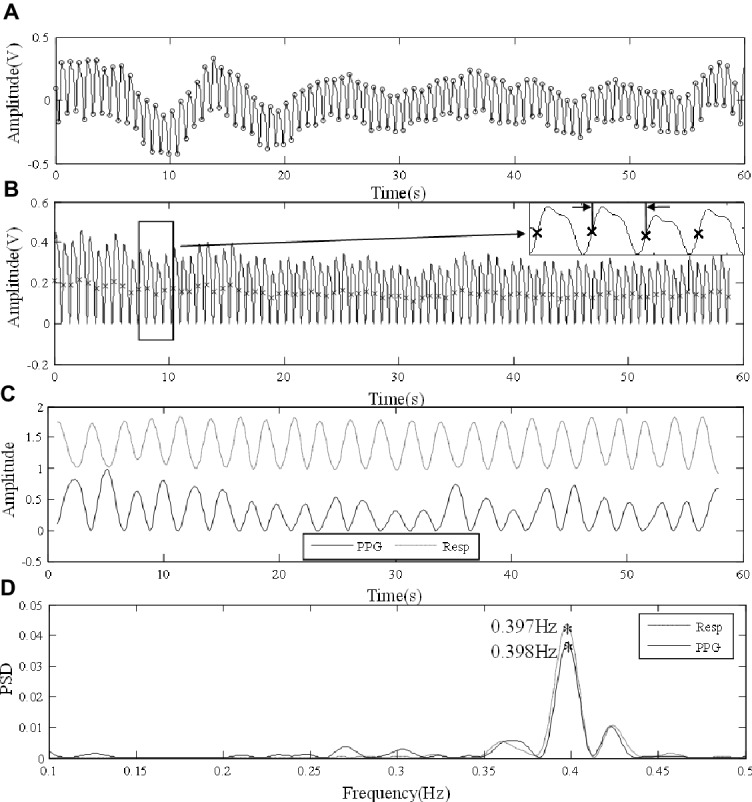
Signal processing procedure of extracting RF from the finger PPG signal of a subject under normal breathing. **(A)** Raw PPG waveform derived from the finger. **(B)** Foot point unification, with the points of maximum slope marked in the locally magnified view. **(C)** The PPG-derived (PPG) and reference (Resp) respiratory signals. **(D)** The extraction of RFs from the respiratory signals by PSD.

Figure 2 illustrates the RF extraction process. First, from low-pass filtered PPG signal, local maximum and minimum values within each cardiac cycle were identified by detecting the change in sign of slope, with minimum distance between consecutive peaks or feet as 0.4 s (Figure 2A). Second, the end-of-diastole foot points of cardiac cycles were unified to zero. First, end-of-diastolic foot points were used to divide the waveform into different cardiac cycles. A piecewise function was then derived by connecting the foot points of consecutive cardiac cycles with segments. Finally, by subtracting the piecewise function from the waveform, the foot points were unified to zero. The maximum slope point of each cardiac cycle was then determined by maximum of its derivative (magnified in Figure 2B). Subsequently, the intervals between PPG pulses were calculated from the maximum slope points. Based on pulse interval modulation (PIM), FM method was used to extract the respiratory signal from the interval variability between pulses (Mukhtar, 2004). The respiratory signals derived from the PPG signals by FM and the reference respiratory signals measured from the chest strap are juxtaposed in Figure 2C. The PPG-derived respiratory signal has been normalized to the range 0–1. The reference respiratory signal was also processed and transformed. They were juxtaposed to illustrate the variations in waveform. Finally, the power spectral density (PSD) was used to extract the RFs from the PPG-derived and reference respiratory signals by selecting the peak of the frequency distribution. PSD was calculated based on Fast Fourier Transform (FFT) using the periodogram function in Signal Processing Toolbox of MATLAB (R2018a; The MathWorks Inc., Natick, USA). In Figure 2D, the corresponding RFs were determined as the main frequencies, both approximately at 0.4 Hz.

### Statistical Analysis

Statistical analysis was performed on SPSS (Version 24.0, IBM Corp). The mean and standard deviation (SD) of the PPG-derived and reference RFs were first calculated across all subjects, separately for different measurement sites and for both breathing patterns. Second, the bias of PPG-derived RFs in comparison with the reference RF was calculated and illustrated separately for different measurement sites and for both breathing patterns. Levene’s test was performed to investigate the homogeneity of variance (defined as *p* more than 0.05). Besides *z* scores of skewness and kurtosis, Kolmogorov-Smirnov test was performed to investigate the normality of data distribution (defined as *p* more than 0.05). For the data with normal distribution and homogeneity of variance, analysis of variance (ANOVA) with least significant difference (LSD) *post hoc* multiple comparisons was then performed to examine if there were significant bias (defined as *p* less than 0.05) between the PPG-derived and reference RFs. Else, Wilcoxon signed rank test was performed to investigate if there were any significant differences (*p* less than 0.05) between PPG-derived and reference RFs if homogeneity of variance was not satisfied. Then Kruskal-Wallis test was performed to investigate whether the RF bias was significantly different (*p* less than 0.05) among body sites or not. The Bland-Altman and linear regression analyses were also performed between the PPG-derived and reference RFs with the slope of best-fit line and corresponding *R*^2^ obtained. To estimate the agreement between PPG-derived and reference RFs, Lin’s concordance correlation coefficient was calculated between the RFs. Because RF was paced at a fixed rate of 0.1 Hz during deep breathing, the expected PPG-derived RF would be within a very narrow range. The linear regression analysis was therefore only performed for RFs under normal breathing but not for the deep breathing condition.

## Results

### Overall Estimation Accuracy of Photoplethysmography-Derived Respiratory Frequency

**Figure 3:**
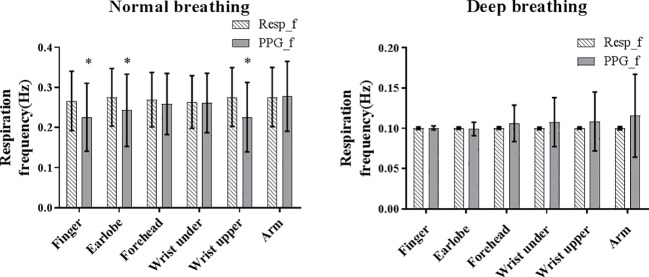
The mean (shaded bar) and standard deviation (segment) of reference (Resp_f) and PPG-derived (PPG_f) RFs under normal and deep breathing conditions. Asterisk marks the significant bias between Resf_f and PPG_f.

Figure 3 shows the means and SDs of the PPG-derived and reference RFs under both normal and deep breathing patterns. Homogeneity of variance was not satisfied by the overall distribution of RF bias (*p* < 0.001 for Levene’s test); therefore, Kruskal-Wallis test was performed. The results showed that RF bias was significantly influenced by the breathing pattern and measurement site (both *p* < 0.001).

Under normal breathing, the overall reference RF was approximately 0.27 Hz from all the measurement sites. The between-subject variations of the PPG-derived RF (SD across all subjects) were comparable between the measurement sites. Homogeneity of variance was satisfied by PPG-derived RF, reference RF, and RF bias (all *p* > 0.05 in Levene’s test). The normality of data distribution was satisfied by PPG-derived RF, reference RF, and RF bias in each body site (*z* score < 3 for skewness and kurtosis, and *p* > 0.05 for all in Kolmogorov-Smirnov test). Therefore, ANOVA was performed. The bias between the PPG-derived and reference RFs was insignificant for the measurements from arm, forehead, and wrist under (all *p* > 0.05) and was significant for the finger, earlobe, and wrist upper (all *p* < 0.05). There was a significant difference in RF bias among all the measurement sites (*p* < 0.05).

Under deep breathing, the calculated mean reference RF was 0.103 Hz as expected due to the application of paced breathing at 0.10 Hz. Homogeneity of variance was not satisfied by PPG-derived RF, reference RF, or RF bias (*p* < 0.01 for all); therefore, Wilcoxon signed rank test and Kruskal-Wallis test were performed. Wilcoxon signed rank test showed that bias between the PPG-derived and reference RFs was insignificant for all measurement sites (all *p* > 0.05), except forehead (*p* = 0.048). Kruskal-Wallis test showed that there was no significant difference in RF bias among all the measurement sites (*p* > 0.05).

### Bland-Altman Analysis: The Estimation Accuracy of the Photoplethysmography-Derived Respiratory Frequencies in Different Sites

**Figure 4:**
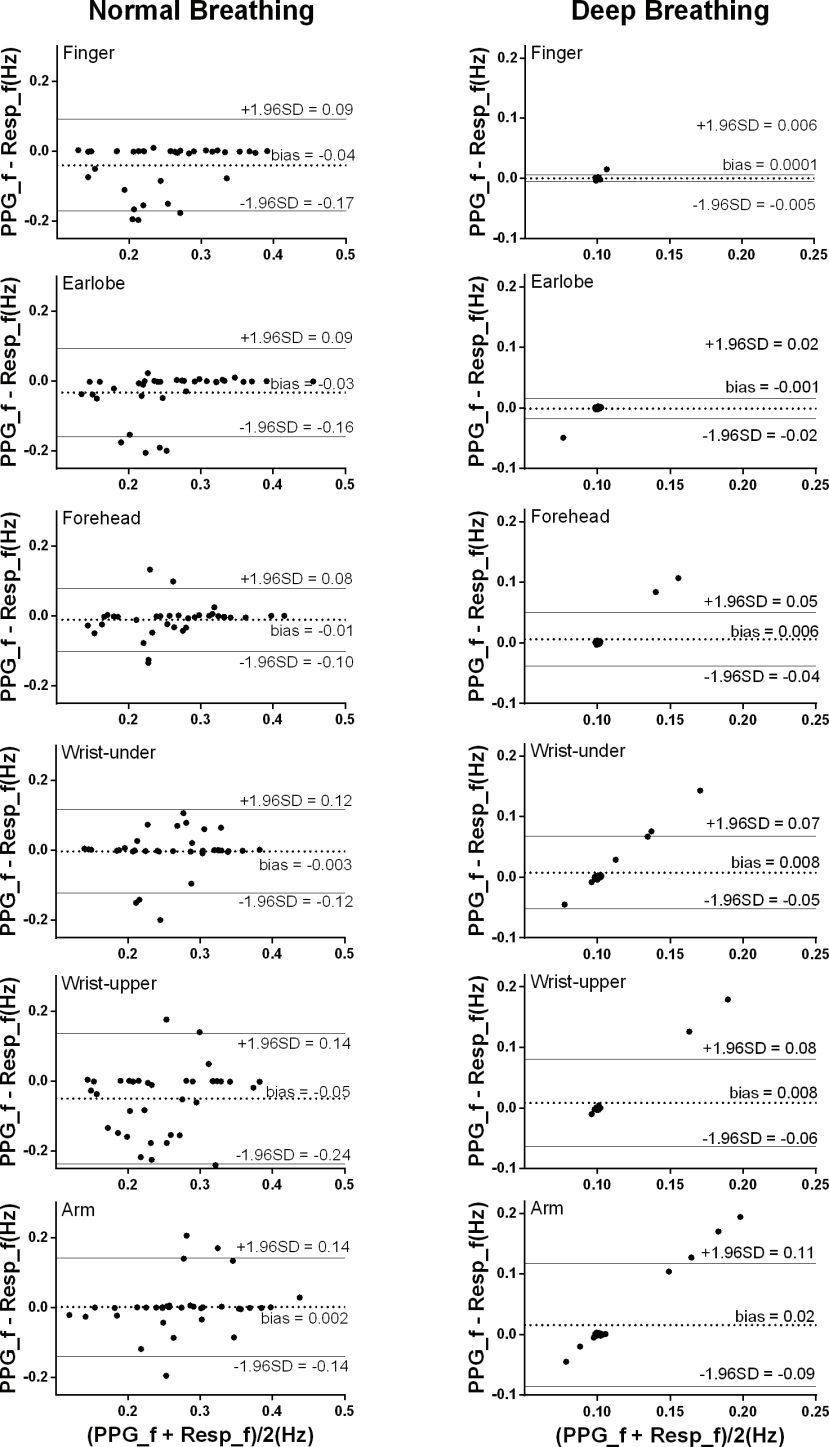
Bland-Altman results of the PPG-derived and reference RFs. The *x* and *y* axes indicate the mean and difference of the PPG-derived and the corresponding reference respiratory frequencies, respectively. The dotted lines show the mean bias. The limits of agreement (LoA) mean ± 1.96 SD interval of difference are given with the solid lines.

As shown in Figure 4, under normal breathing, the smallest bias between the PPG-derived and reference RF was from the measurement of the arm (bias: 0.0018 Hz, 95% limits of agreement (LoA): −0.14 to 0.14 Hz), followed by wrist under, forehead, earlobe, finger, and wrist upper where the bias and 95% LoA were −0.05 Hz and −0.24 to 0.14 Hz, respectively. The estimation bias was between −0.011 and −0.05 Hz in all sites. In the *post hoc* test of ANOVA on RF bias, the forehead was significantly different from wrist upper (*p* < 0.05). The numbers of outliers beyond 0.1 Hz were within five for forehead and earlobe. Forehead had the narrowest LoA (−0.10 to 0.08 Hz), indicating the best performance of the PPG-derived RF estimation.

Under deep breathing, the overall bias (ranged between 0.00026 and 0.016 Hz) was much smaller than the results from normal breathing. The smallest bias between the PPG-derived and reference RFs was from the finger measurement with the narrowest LoA (bias: 0.00026 Hz, 95% LoA: −0.0054 to 0.006 Hz), followed by earlobe, forehead, wrist upper, wrist under, and arm (bias: 0.016 Hz, 95% LoA: −0.086 to 0.12 Hz).

### Linear Regression Analysis Between the Photoplethysmography-Derived and Reference Respiratory Frequencies

**Figure 5:**
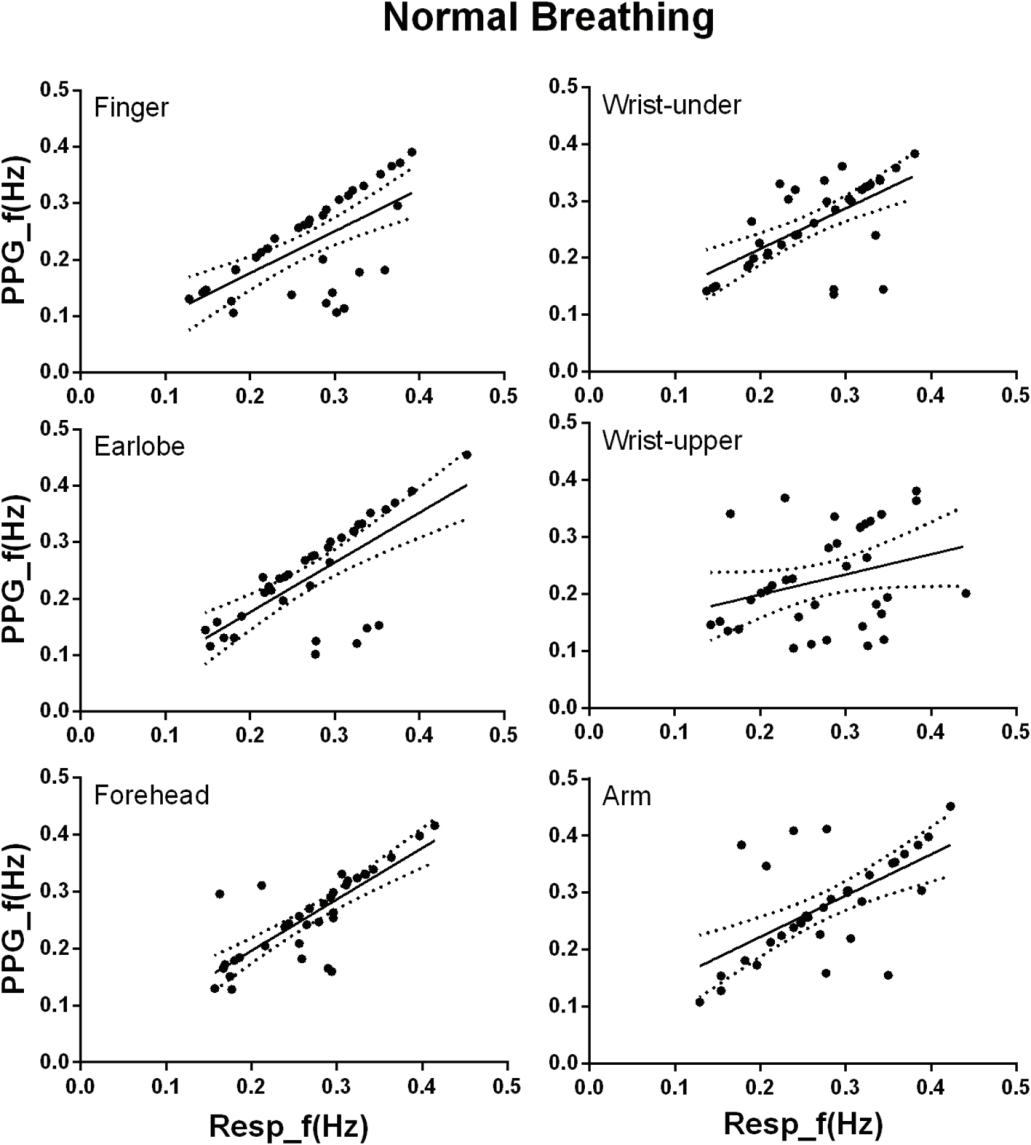
Linear regression of the PPG-derived and reference RFs under normal breathing. The *x* and *y* axes denote the reference and PPG-derived RFs, respectively. The points mark the measured data. The solid and dotted lines indicate the best-fit line and its 95% confidence interval, respectively.

Under normal breathing, there was significant linear relationship between the PPG-derived and reference RFs for the measurements from all the sites (all *p* < 0.001, except for the wrist upper, *p* = 0.07; Figure 5). According to the regression line slope and correlation coefficient, the overall agreement between the PPG-derived and reference RFs was best at the forehead (slope of best-fit line: 0.90, *R*^2^: 0.64), followed by the earlobe (0.88, 0.50), finger (0.74, 0.42), finally the arm (0.73, 0.37), and wrist under (0.71, 0.40). Additionally, Lin’s concordance correlation coefficient was the highest at the forehead (0.788), followed by the earlobe (0.635), arm (0.602), wrist under (0.593), finger (0.568), and finally the wrist upper (0.221). Therefore, the best agreement between PPG-derived RF and reference RF was at the forehead.

## Discussion

This study quantitatively investigated the accuracy of RF extraction from the PPG signals measured from different body sites. To the best of our knowledge, this is the first comprehensive comparison of the accuracy of the PPG-derived RF between different body sites, with the consideration of different breathing patterns. In comparison with the reference RF, the best PPG-derived RF was obtained from the forehead and finger, respectively, for normal and deep breathing patterns.

Under normal breathing, it could be concluded from this study that PPG measurement from forehead could be used to achieve the best RF extraction. The RF bias was insignificant in arm, wrist under, and the forehead (*p* > 0.05). The highest *R*^2^ and the highest Lin’s concordance correlation coefficient between the PPG-derived and reference RFs and the narrowest LoA were also observed in forehead. The relatively better RF estimation from the forehead could be explained by: first, it has been reported that the PPG signal from forehead has much stronger respiratory component than other sites (Shelley et al., 2006). Second, in peripheral arteries, vasoconstriction and other compensatory mechanisms retaining arterial BP influence PPG signals, with delayed observations of hypoxemia and blood loss (McGrath et al., 2011; Ermer et al., 2017). The forehead is the only microcirculation in the entire skin that has no vasoconstriction properties and is supplied by the internal carotid artery. Therefore, PPG signal from forehead is the most sensitive to the temporal fluctuation of blood oxygen saturation (MacLeod et al., 2005; Bebout and Bednarski, 2017), enabling the best RF estimation.

Under deep breathing condition, finger PPG measurements produced the smallest RF bias and the smallest LoA. Firstly, in deep breathing, the prolonged respiratory cycle decreases the difference between sites in the ability of reflecting the oxygen saturation, weakening the relative superiority of forehead PPG signal. The nadir of blood oxygen saturation needs 5 and 15 s to be reflected in the PPG signals of ear and finger (Lindholm et al., 2007). The prolonged respiratory cycle enables the PPG signals of other sites besides forehead to follow the fluctuations of blood oxygen saturation. Secondly, considering the similar ability of PPG signals from different sites in following the oxygen saturation, the accuracy of the resultant PPG-derived RFs therefore depends more on the quality of PPG signal itself. Finger was suggested as the best measurement site of high-quality PPG signal due to the rich arterial supply and the relative convenience to affix sensors (Sun and Thakor, 2016). Thirdly, due to the blood supply by intracranial arteries, the PPG signal from forehead is strongly influenced by other physiological signals, especially the Mayer waves compared with other sites (Pfurtscheller et al., 2018). The frequency ranges of different physiological information are partly superimposed instead of strictly separated, which might cause PPG waveform change and consequent inaccuracy in RF extraction by FM. It has been reported that the noises of 0.1–0.2 Hz affected the effectiveness of RF extraction from the PPG signal of head, while the signal from finger was nearly free from these noises (Hernando et al., 2017). In Figure 4, under deep breathing, the RF bias was obvious at about 0.15 Hz at forehead, but unobvious at the finger and earlobe. Finally, with FM method, respiratory signals were of higher quality when extracted from finger PPG than ear PPG (Charlton et al., 2017), which is more affected by the motion noises (Bentham et al., 2018). In Figures 3, 4, the finger shows smaller RF bias than the ear.

## Explanations of Estimation Bias in This Study with Examples

It has been observed that there were two major factors that were related to the estimation errors in this study. They were the PPG signal quality and the varying breathing pattern.

**Figure 6:**
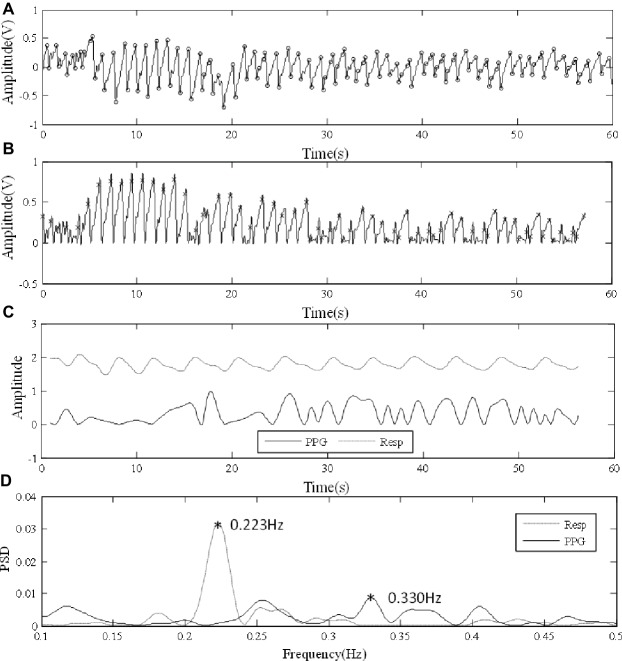
Error in analysis due to low-quality PPG signal. **(A)** Low-quality PPG waveform from wrist under normal breathing. **(B)** Irregular amplitude and frequency fluctuations after foot point unification. **(C)** Comparisons between PPG-derived and reference respiration signals. **(D)** PSD results.

First, the accuracy of the PPG-derived RF depends on the quality of the raw PPG signal. Figure 6A shows a low-quality raw PPG waveform from wrist under normal breathing. Wrist is a site where a reliable and clear PPG signal is difficult to be measured accurately (Geun et al., 2008). As shown in Figure 6B, possibly due to motion artifact, local fluctuations caused erroneous detection of multiple local peaks and feet in one cardiac cycle, resulting in inaccurate cardiac cycle division. Consequently, the PPG-derived respiratory signal was non-periodic and obviously different from the reference respiratory signal (Figure 6C). There was no clear frequency peak in the derived respiration signal, as shown in Figure 6D, resulting in the measurement error larger than 0.1 Hz. Similarly, the two outliers in the forehead results under deep breathing in Figure 4 were due to inaccurate positions of maximum slope points caused by the noisy PPG signal. Therefore, it is important to improve the robustness of RF algorithm for low-quality PPG signals.

**Figure 7:**
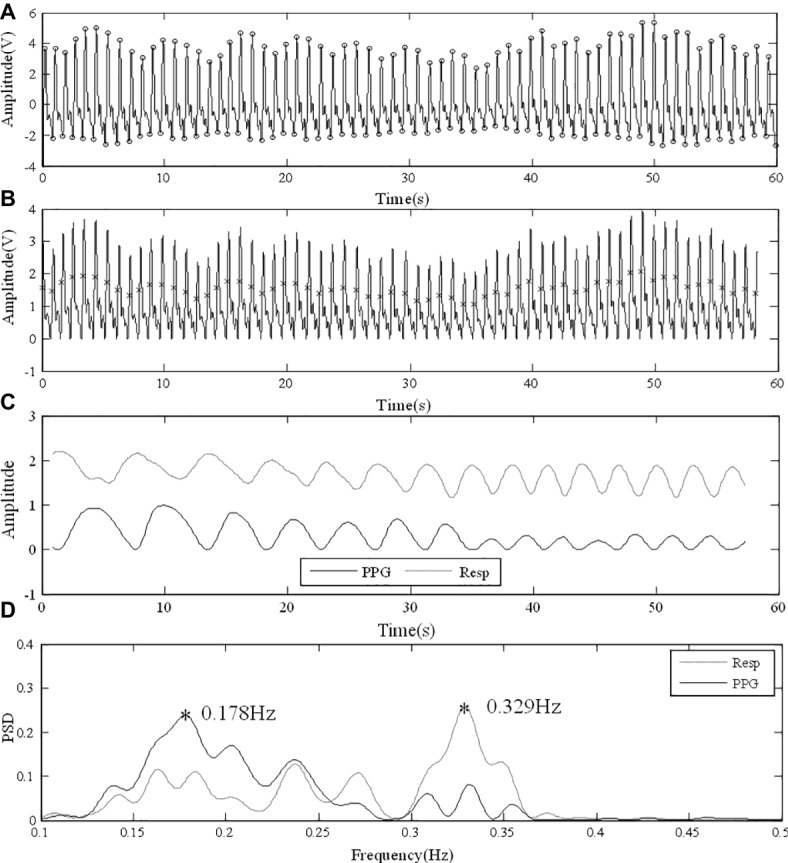
Error in analysis due to the transition between different RFs. **(A)** Raw PPG signal from finger under normal breathing. **(B)** Foot point unification. **(C)** PPG-derived and reference respiratory signals with similar fluctuating patterns. **(D)** PSD results with different RF peaks detected.

The transition between breathing frequencies could also affect the PPG-derived RF. In Figure 7A, the subject’s breathing was regular for the first 30 s and then changed and ended up with a fast breathing. The finger PPG signal under normal breathing was clear and enabled the extraction of reliable respiratory signal (Figure 7B). Figure 7C shows the similar patterns of PPG-derived and reference respiratory signals, with different amplitudes, especially in the later period of rapid respiration. The local peaks or inflection points of the reference respiratory signal are reflected in the PSD result of the PPG-derived respiratory signal. However, due to the aliasing between the RFs of the two periods, the detection of frequency peak was affected in the PPG-derived signal. In Figure 7D, in the PPG-derived results, the global frequency peak was erroneously detected at 0.178 Hz instead of 0.329 Hz as in the reference signal. During measurement, despite enough resting time, it was difficult for each subject to reach steady breathing pattern. Furthermore, it is common that RF has minor variations due to various physiological activities during measurement. Therefore, further investigations are needed to improve the algorithm to detect RF variations without being affected by harmonics.

## Limitations and Future Directions

Limitations of this study include, first, only FM was used for RF extraction. The detection of RF from PPG signal by FM is difficult in some clinical conditions, where the respiratory arrhythmia is strongly altered, such as frequent premature beats, the presence of pace maker, heart transplantation, and severe dysautonomia. The combination of FM, AM, and BW could improve the accuracy and robustness of the RF extraction (Pimentel et al., 2017). Additionally, the sensitivity to motion artifact on the PPG-derived RF could be reduced by the combination with accelerator for measuring the movements (Shen et al., 2017). Furthermore, algorithmic improvement on the detection of maximum and minimum points could improve FM accuracy by avoiding the erroneous cardiac cycle division as shown in Figure 6 and the inaccuracy in detecting maximum slope points, which led to the outliers of Figure 4. Nevertheless, our study focused on providing a primary comparison between PPG measurement sites in deriving RF based on a commonly used method. Second, in order to achieve a controlled comparison between measurement sites, only the traditional infrared light source was used for PPG measurements in this study. It has been shown that the green light could be more suitable for the wrist measurement, and an infrared light source is better for the forehead PPG (Vizbara, 2013). The interaction between RF extraction from different measurement sites, and the use of different light sources needs further investigation. Finally, the measured RF was within the normal physiological range from healthy subjects in supine position. It has been reported that algorithms for extracting RF from PPG signals are inaccurate when RF is outside the normal physiological range (e.g., higher than 0.5 Hz; Nam et al., 2014; Lázaro et al., 2015). Especially, FM method is based on the respiratory sinus arrhythmia, which decreases at high RF. Therefore, estimation of high RF will be difficult by FM and might need the combination of AM and BW. Additionally, RF was controlled (0.1 Hz) under deep breathing, data collection under uncontrolled deep breathing could be performed to further investigate the effect of interaction between body site and breathing pattern on PPG-derived RF. In future work, more advanced RF-extraction methods, diverse light sources, different postures such as standing and sitting, subjects under different physiological and pathological conditions could be considered, with more detailed statistical analysis based on parameters such as accuracy, sensitivity, and specificity.

In conclusion, this study quantitatively compared the estimation accuracy of the PPG-derived RF from different body sites and demonstrated that the estimation accuracy depends on the measurement site and breathing pattern. The best sites are the forehead and finger, respectively, for normal and deep breathing conditions, providing a scientific evidence for future work on the estimation of respiratory information from PPG signals.

## Ethics Statement

This study was carried out in accordance with the recommendations of the Local Research Ethics Committee of the Faculty Research Ethics Panel (FREP) under the terms of Anglia Ruskin University with written informed consent from all subjects. All subjects gave written informed consent in accordance with the Declaration of Helsinki. The protocol was approved by the Local Research Ethics Committee of the Faculty Research Ethics Panel (FREP) under the terms of Anglia Ruskin University.

## Author Contributions

VH performed most of the original experiments described in this study. HL, WH, and VH analyzed the data. All authors contributed to the discussion. HL finished the draft of manuscript. DZ and FC supervised the project that led to production of the results shown here; moreover, they critically reviewed and edited the manuscript. All authors concur with the current submitted version.

### Conflict of Interest Statement

The authors declare that the research was conducted in the absence of any commercial or financial relationships that could be construed as a potential conflict of interest.
